# Changing Dietary Habits in Veneto Region over Two Decades: Still a Long Road to Go to Reach an Iodine-Sufficient Status

**DOI:** 10.3390/nu12082399

**Published:** 2020-08-11

**Authors:** Simona Censi, Jacopo Manso, Susi Barollo, Alberto Mondin, Loris Bertazza, Massimo De Marchi, Caterina Mian, on behalf of the Food and Nutrition Hygiene Services (SIAN)

**Affiliations:** 1 Department of Medicine (DIMED), Endocrinology Unit, University of Padova, 35121 Padova, Italy; simona.censi@phd.unipd.it (S.C.); jacopo.manso@gmail.com (J.M.); susibarollo@yahoo.it (S.B.); alberto.mondin.1@studenti.unipd.it (A.M.); loris.bertazza@unipd.it (L.B.); 2 Department of Agronomy, Food, Natural Resources, Animals and the Environment (DAFNAE), University of Padova, 35020 Legnaro (PD), Italy; massimo.demarchi@unipd.it

**Keywords:** iodine, milk, iodized salt, dairy products, eating habits

## Abstract

**Background:** Fifteen years after a nationwide voluntary iodine prophylaxis program was introduced, the aims of the present study were: (**a**) to obtain an up-to-date assessment of dietary iodine intake in the Veneto region, Italy; and (**b**) to assess dietary and socioeconomic factors that might influence iodine status. **Methods:** Urinary iodine concentration (UIC) was obtained in 747 school students (median age 13 years; range: 11–16 years). **Results:** The median UIC was 111 μg/L, with 56% of samples ≥ 100 μg/L, but 26% were < 50 μg/L, more frequently females. Iodized salt was used by 82% of the students. The median UIC was higher among users of iodized salt than among non-users, 117.0 ug/L versus 90 ug/L (*p* = 0.01). The median UIC was higher in regular consumers of cow’s milk than in occasional consumers, 132.0 μg/L versus 96.0 μg/L (*p* < 0.01). A regular intake of milk and/or the use of iodized salt sufficed to reach an adequate median UIC, although satisfying only with the combined use. A trend towards higher UIC values emerged in regular consumers of cheese and yogurt. **Conclusion:** Iodine status has improved (median UIC 111.0 μg/L), but it is still not adequate as 26% had a UIC < 50 μg/L in the resident population of the Veneto region. A more widespread use of iodized salt but also milk and milk product consumption may have been one of the key factors in achieving this partial improvement.

## 1. Introduction

Iodine deficiency (ID) affects two billion people around the globe and is the world’s greatest single cause of preventable brain damage [1]. A low iodine intake in fetal life and early infancy can interfere with adequate brain development and growth, and a moderate-to-severe deficiency can reduce the intelligence quotient (IQ) by 10–15 points in childhood [2,3]. The consequences of a mild-to-moderate ID are less clear, but many studies suggest that it can prevent children from reaching their full intellectual and verbal potential [4,5,6]. In the last decade, numerous strategies have been implemented around the world to prevent ID, including Universal Salt Iodisation (USI). In measuring the effectiveness of a USI program in eliminating ID from a population, the World Health Organization (WHO) recommends that more than 90% of households should be using iodized salt. In addition, the median Urinary Iodine Concentration (UIC) of the population must fall within the adequate range of 100–199 μg/L, with no more than 20% with a UIC < 50 μg/L [1].

When the physiological requirements of iodine are not met in a given population, developmental and functional abnormalities occur, including thyroid function anomalies and, when ID is severe, endemic goiter and cretinism [7]. An area is considered to be affected by endemic goiter when more than 5% of school-age children (SAC) have an enlarged thyroid, according to the WHO criteria [1], although a worldwide standardization of the ultrasound technique of thyroid volume measurement between groups working in the field of ID would be needed [8]. The median UIC established from spot urine samples obtained from representative cohorts of SAC is the parameter most often used to classify a population’s iodine status. 

As Italy has historically been characterized by endemic goiter [7], a nationwide voluntary iodine prophylaxis program was implemented in 2005 (in law n.55/2005). As a consequence of this law, iodized salt became available in each food store, with non-iodized salt accessible only on specific costumers’ request. Moreover, iodized salt was available in all bars, restaurants, collective caterings and canteens and its use was authorized in processed food preparation.

In addition to the national voluntary iodine prophylaxis program, since 2013, the Italian Ministry for Education, University, and Research (MIUR) has been running a nationwide education program on the importance of iodine for human health and the benefits of using iodized salt use in all secondary and higher-level schools, with the aim of improving iodine nutrition. The educational program on iodine prophylaxis in the Veneto region consists of several steps. Physicians from our Endocrinology Unit provide training for the medical staff at the Food Hygiene and Nutrition Services (SIAN) with the aid of several computer-based materials provided by the MIUR illustrating the main concepts behind iodine prophylaxis and the importance of a responsible use of salt. Then the SIAN staff train the schoolteachers at the designated schools, and the teachers in turn conduct educational sessions with their students, using targeted oral presentations, based on a multistep program already used in a previous survey [9].

Prior to the introduction of 2005 voluntary iodine prophylaxis program, iodine status assessments conducted between 1994 and on SAC living in north-east Italy demonstrated a non-negligible prevalence of thyroid goiter (on physical examination, 7.5% of children had grade I goiter [10]), and iodized salt was used by less than 30% of the SAC [6]. Nevertheless, median UIC values showed iodine sufficiency (median UIC of 140 μg/L on data collected in 2001), thanks to the extensive consumption of milk (about 70% of SAC took 200 mL of milk or more per day) [6]. The pretty high prevalence of goiter, a parameter of long-term iodine status, suggested that this result was not reflecting a long-term iodine-sufficiency, but rather a recently obtained goal.

In 2012 [11], seven years after starting the iodine prophylaxis program, the use of iodized salt had spread to 70% of the Veneto region’s households, but the median UIC in the population fell in the range of mild-to-moderate iodine deficiency (81 ug/L, 95%CI 74–87 μg/L). The change in dietary habits, with a gradual reduction of milk and dairy consumption, a phenomenon observed in Veneto region but also in other developed areas in the world [12], has prevented Veneto to reach an iodine sufficiency despite USI. Across the surveys conducted from 1994 to 2012 the eating habits of SAC have shown gradual changes, with a decline in the consumption of milk, yogurt, and cheese, from 69% to 46.7%; from 48% to 28.6%; and from 65% to 44.2%, respectively [6,11]. The findings of the survey in 2012 showed that only a combination of iodized salt use and daily milk consumption was needed to achieve an adequate iodine intake.

The influence of socioeconomic and educational factors on voluntary iodine prophylaxis program is presented. In many regions of the world, a low education level proved a predictor of reduced efficacy of such programs [13,14]. In European countries, data on the association between socioeconomic status, education, and iodine supply are more conflicting [15,16]. Moreover, as regards nationality, in a previous report by our group, on data collected between 2015 and 2016, we reported a higher receptiveness of foreign-origin SAC towards the educational intervention, when compared to Italian-origin SACs [9].

Preliminary data from the Italian National Observatory for Monitoring Iodine Prophylaxis (OSNAMI) for the years 2015–2019, on a sample of 2523 SAC (11–13 years old) residing in 7 Italian regions (Liguria, Toscana, Emilia Romagna, Marche, Umbria, Lazio, Sicilia), indicated that the UIC was now sufficient (118 ug/L), and iodized salt was used in 75% of Italian school canteens. The Veneto region was not among the regions included in this preliminary analysis [17].

The aims of the present study were: (**a**) to conduct an up-to-date assessment of iodine status in our Veneto region; and (**b**) to examine dietary and socioeconomic factors that might influence it.

## 2. Patients and Methods

The periodic monitoring of iodine status among SACs is included in OSNAMI tasks and Padua is the reference centre for iodine prophylaxis in the Veneto region. SIAN periodically indicates schools to be enrolled, representative of Veneto region. Ten schools, distributed in all Veneto provinces and territories (mountain, hills, and lowland), were selected. These schools represent the 1.7% of middle schools in Veneto region (10/578). This study was conducted in accordance with the Declaration of Helsinki. For all participants, verbal informed consent was obtained from the parent/guardian on behalf of the minor, witnessed, and formally recorded. The investigation was conducted between September 2018 and September 2019 on 11- to 16-year-old SAC in grades 6 to 8. To survey the students’ awareness about iodine prophylaxis and iodine status, we enrolled students living in 10 different cities located in all 7 provinces of the Veneto region. Pupils attending middle schools adhering to the iodine prophylaxis monitoring program were asked to participate to the investigation.

Pupils were requested to complete a questionnaire, with the help of their parents and to collect a non-fasting early-morning spot urine sample. The inclusion criteria were an obtained informed consent, a fully completed questionnaire and urine samples properly collected. The questionnaire comprised two sections: one recorded personal socioeconomic information (including nationality, and parents’ education level), any personal history of thyroid diseases, and how much students knew about iodine prophylaxis; the other addressed their dietary habits, and particularly their consumption of the following products: cow’s milk, dairy products, yogurt, beef, chicken/turkey, eggs, fish, canned food, cold cuts, soft drinks, chips, soy products, and sodium-rich foods (pizza, soda, and chips).

These types of food were chosen to obtain a measure of the amount of iodine deriving from different iodine sources: from naturally-rich in iodine food (fish); from the policy of cattle food iodine enrichment and breeding’s diet in general (milk, yogurt, and cheese, but also eggs, beef, chicken, and turkey meat, although the latter are marginal sources of iodine). Since law n.55/2005 encourages the use of iodized salt in food preparation and conservation, also the contribution deriving from industrial food processing was considered, including prepackaged food (canned food, soy products, and soft drinks), processed food (cold cuts), and sodium-rich foods (pizza and chips).

Nationality was dichotomized in Italian or foreign.

Parental education level was recorded as the highest qualification obtained (university degree, high-school diploma, middle-school diploma, or elementary school). The total number of years of education completed was also recorded, with the data considered for each parent separately and also as both parents summed.

The knowledge about iodine prophylaxis campaign was assessed by asking if the pupils already knew about the program and about the existence of a legislation regulating that and from what information source (school educational program, mass medias, healthcare providers, and institutions).

As regards iodized salt use, pupils’ parents were asked if he/she used iodized salt (yes/no) and for how many years. 

Cow’s milk intake was scored as: 1 = no consumption; 2 = occasional consumption; 3 = 1 cup a day (at least 200 mL/d); and 4 = more than 1 cup a day. Cow’s and soy milk consumption was also dichotomized as: 1 = regular (daily or more than once a day) or 0 = occasionally (less than once a day). For other foods, intake was scored as: 1 = never; 2 = at least once a week; or 3 = more than once a week.

A 20 mL sample of early-morning urine in non-fasting conditions was collected from all students. Samples were divided into aliquots and refrigerated at –20 °C until assay. UIC was expressed as μg/L and measured in duplicate using the colorimetric ceric ion arsenious acid method in a second-analyzer Technicon Auto-Analyzer (Brain Luebbe GmbH, Norderstedt, Germany) [18]. Between-run inaccuracy was assessed by repeatedly measuring two levels of in-house quality control material (95 and 300 μg/L), and was from 1.7% to 2.8%. At the start of each run, a standard curve was also recorded with serial dilutions of a synthetic tester [9].

### Statistical Analysis

The Kolmogorov–Smirnov test was used to check the normal distribution of the UIC. As the values were not distributed normally, they are reported here as medians and interquartile range (IQR). The Mann–Whitney U test was consequently used to analyze the differences in UIC in relation to different variables, when dichotomized. The Kruskal–Wallis test was used to study possible changes in median UIC when more than two possible scores of a given variable were taken into account. The χ2 test was used to explore the relationship between different iodine settings (< 50 μg/L, 50 ≤ UIC < 100 μg/L, and ≥ 100 μg/L) and dietary and socioeconomic factors (nationality, parents’ educational level). Univariate analysis (by logistic regression) was used to explore the relative influence of each independent variable on the UIC. A *p* value of < 0.05 was considered statistically significant. 

## 3. Results

### 3.1. Iodine Status

**Figure 1:**
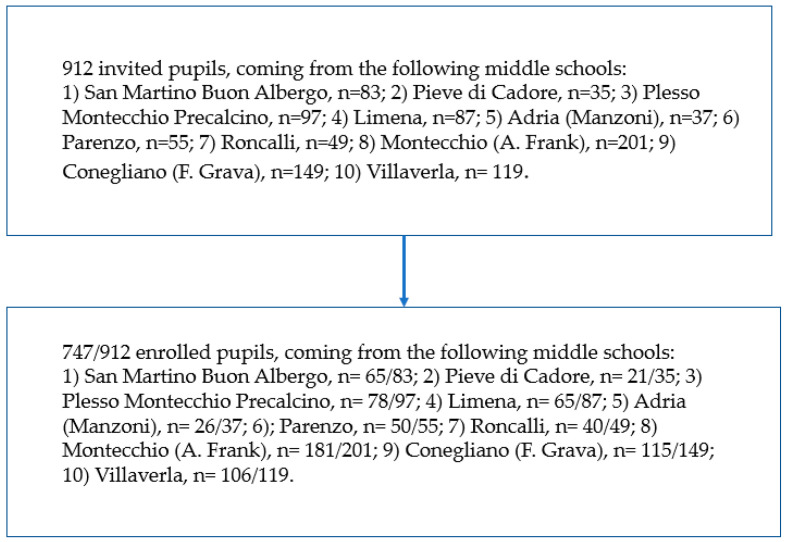
Flow-chart representing enrollment model.

Nine hundred and twelve pupils were invited. Seven hundred and forty-seven pupils complied with the inclusion criteria and were thus included in the study (747/912, 82%) (Figure 1). This pool of students represents the 0.6% (747/134,907) of the totality of pupils attending middle schools in Veneto region. The median age was 13 years (range 11–16 years), 411 (55%) were males, and 336 (45%) were females. Data from all 747 enrolled students were included. The median UIC in the SAC enrolled was 111.0 ug/L (IQR: 55.9–198.0 μg/L). The value was higher in males than in females, being 121.0 μg/L (IQR: 56.4–199.2 μg/L) in the former and 103.2 μg/L (IQR: 37.2–174.0 μg/L) in the latter (*p* < 0.01) (Table 1).

Table 2 shows the percentage of samples with a UIC ≥100 μg/L, between 50 μg/L and <100 μg/L and <50 μg/L) and the corresponding median UIC in the whole sample, according to iodized salt use and food consumption frequency. As shown in the Table 1, the UIC was ≥100 μg/L in 56% of the students (416/747), between 50 and <100 μg/L in 18% (138/747), and <50 μg/L in 26% (193/747).

In Table 1 UIC median values and IQR and the % of pupils with a UIC < 50 μg/L, based on sex and on age group are given. A percentage of 23.6% (97/411) of males and of 30.3% (102/336) of females had a UIC < 50 μg/L. 11–12-years old and 15–16-years old females resulted as iodine-deficient.

### 3.2. Dietary Habits

#### 3.2.1. Iodized Salt

Eighty-two percent (610/747) of the students interviewed used iodized salt. Iodized salt was used by 336/411 (81.8%) of males and by 279/336 (83.0%) of females. The median UIC was higher among students using iodized salt than among non-users, being 117.0 μg/L (interquartile range: 54.0–192.0 μg/L) in the former and 90.0 ug/L, (interquartile range: 36.6–174.6 μg/L) in the latter (*p* = 0.01) (Table 2). The students’ households had been using iodized salt for at least 10 years in 75% of cases. The students living in families where iodized salt had been used for at least 10 years had a higher median UIC, being 127.8 μg/L (IQR: 57.6–192.0 μg/L) as opposed to 97.2 μg/L (IQR: 48.0–177.6 μg/L) in the remainder of the sample, although the difference was not statistically significant (*p* = 0.15). The percentage of students with UIC values ≥ 100 μg/L was also higher among the families using iodized salt for more than a decade (61% as opposed to 49% in the remainder of the sample), although this difference did not reach statistical significance (*p* = 0.07).

#### 3.2.2. Cow’s Milk

Daily cow’s milk consumption was reported by 47% (349/474) of the students, with a higher percentage among males (54%, 221/411) compared to females (38%, 126/336), *p* < 0.01. The median UIC was higher in regular consumers of cow’s milk than in occasional users, being 132.0 μg/L (IQR: 72.0–198.9 μg/L) in the former and 96.0 μg/L (IQR: 36.0–177.3 μg/L) in the latter (*p* < 0.01) (Table 2). The percentage of students with UIC ≥ 100 μg/L rose gradually and significantly with higher intakes of milk (Table 2).

#### 3.2.3. Cow’s Milk and Iodized Salt

**Figure 2:**
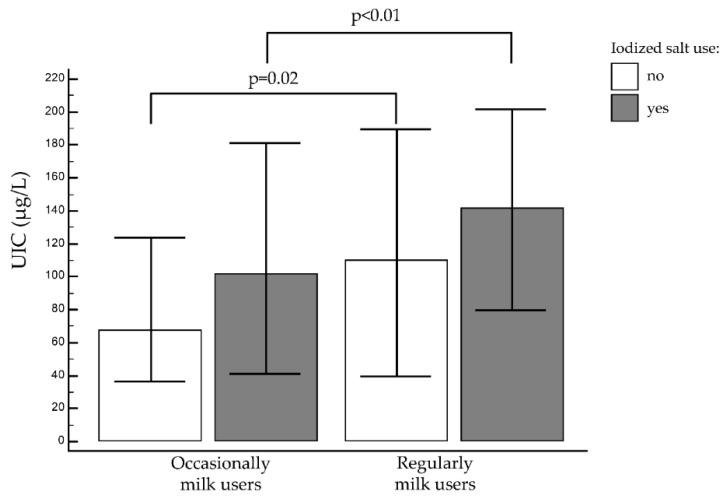
Median urinary iodine concentration (UIC) and milk intake in iodized salt users (grey bars) and non-users (white bars). Bars represent UIC median values (μg/L), while error bars represent interquartile range interval of the median value; milk: occasionally = less than once a day, regularly = daily or more.

Iodized salt users had a sufficient iodine intake, irrespective of their cow’s milk consumption. The median UIC reached 102.0 μg/L (IQR: 41.1–181.50 μg/L) for users of iodized salt who never or only occasionally drank cow’s milk. This level indicates an adequate iodine intake, though it is significantly lower than in iodized salt users who also drank cow’s milk regularly, whose median UIC was 141.0 μg/L (IQR: 79.5–201.3 μg/L), *p* < 0.01 (Figure 2). 

Conversely, students not using iodized salt needed to drink cow’s milk daily to reach an adequate UIC. The median UIC for students not using iodized salt was 67.8 μg/L (IQR: 36.0–123.6 μg/L) among the occasional cow’s milk consumers, and 110.4 μg/L (IQR: 39.3-189.6 μg/L) among the regular cow’s milk consumers, (*p* = 0.02) (Figure 2). 

In short, regular milk consumption and iodized salt use both sufficed alone to reach an adequate median UIC. 

#### 3.2.4. Other Foods, Cheese, and Yogurt

**Figure 3:**
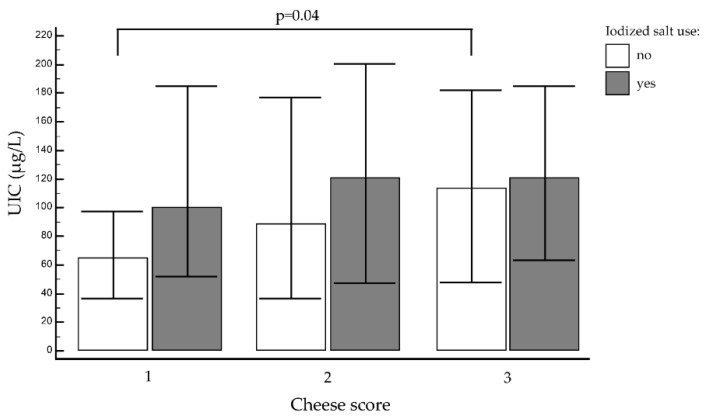
Median urinary iodine concentration (UIC) and cheese score (1 = never; 2 = at least once a week; or 3 = more than once a week) in iodized salt users (grey bars) and non-users (white bars). Bars represent UIC median values, (μg/L), while error bars represent interquartile range interval of the median value.

No association emerged between other foods and median UIC or UIC subgroups. Regular consumers of cheese (more than once a week, 330/747 (44.2%)) did not have higher median UIC than those who ate it less often (never or up to once a week, 89/747 (12%)), however, although the former showed a trend towards higher values (Figure 3). On the other hand, when only the non-iodized salt users were considered, and students eating cheese more than once a week (regular consumers) were compared with those not eating it rarely or not at all, the former had an adequate median UIC, while the latter did not, their median UIC being 114.0 μg/L (IQR: 48.0–182.4 μg/L) and 64.8 μg/L (IQR: 36.0–106.8 μg/L), respectively, *p* = 0.04 (Figure 3).

**Figure 4:**
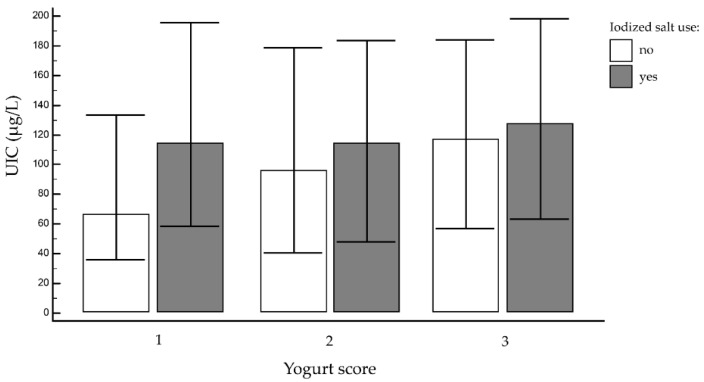
Median urinary iodine concentration (UIC) and yogurt score (1 = never; 2 = at least once a week; or 3 = more than once a week) in iodized salt users (grey bars) and non-users (white bars). Bars represent UIC median values, (μg/L), while error bars represent interquartile range interval of the median value.

Our analysis identified much the same picture for other milk products (yogurt): among the students not using iodized salt the median UIC was 116.4 μg/L (IQR: 56.7–183.9 μg/L) for those who ate yogurt more than once a week (213/747 (28.6%)) and 66.0 μg/L (IQR: 36.0–132.9 μg/L) for those who did not (205/747(27.4%)) (Figure 4), but the difference lacked statistical significance in this case.

#### 3.2.5. Univariate Analysis of Dietary Factors 

The influence of milk on UIC was confirmed by logistic regression model, taking into consideration milk frequency (at least once a day or more versus less than once a day or never), iodized salt use and cheese consumption frequency (more than once a week versus never). Milk frequency has a higher impact on UIC adequacy than iodized salt (milk OR 1.9, 1.4–2.6, iodized salt use, OR 0.69 (0.5–1.0), *p* < 0.01). 

### 3.3. Perception of the Importance of Iodine for Human Health and Relationship with Parent’s Educational Level

In 42.5% of cases (317/747), the students knew there was legislation to deal with iodine deficiency (Italian law n.55/2005). Among these students, those who were aware of the significance of iodine prophylaxis used iodized salt more than those who were not, i.e., 85.6% of the former (271/317) versus 79.5% of the latter (342/430), *p* = 0.04. When students were asked about their main source of nutritional information on iodine, 55% mentioned the mass media (internet, magazines, radio, and television), and only 45% cited the school education program, although it had been running in Italy since 2013. Less than 1% of the sample mentioned their physician or some other institution (e.g., regional or municipal health campaigns).

No association was found between the use of iodized salt or median UIC and the educational level of student’s parents (which ranged from elementary school to a university degree). Nor was there any association between the use of iodized salt or median UIC and student’s nationality (Italian versus others).

## 4. Discussion

A partially adequate iodine status (median UIC of 111 μg/L) has been reached in the population living in the Veneto region since the start of an iodine prophylaxis campaign in 2005 and systematic monitoring of iodine intake in SAC. However, following WHO recommendations, no more than 20% of UIC should be <50 μg/L to define a population iodine-sufficient. Since 26% of our population still has a UIC that falls into a moderate iodine deficiency (<50 μL), much more work should be made in Veneto region to reach iodine adequacy. A more widespread use of iodized salt, if compared to the past consumption in our region [11], is likely to have been one of the key factors in improving iodine status, if compared to the 2012 survey [11]. Though still below the WHO target (90% of households), the percentage of households routinely using iodine-fortified salt has risen from 70% in 2012 [11] to 82% in this last report.

The amount of iodine added to salt established by Italian law is 30 mg per kilo (tolerance range: 24–42 mg/Kg) and was calculated estimating an average consumption of salt of 5 g per day (following WHO recommendations) and the median amount of iodine provided by food in a varied diet [19]. Data on the real salt daily intake among schoolchildren living in our region is still lacking. By the way, a nationwide survey estimated that the recommended iodine intake in children (120 μg/day) is achieved with a daily consumption of just 3 g of iodized salt [20].

In 2012, it was necessary to combine the use of iodized salt with daily cow’s milk consumption to ensure an adequate median UIC in our region [11]. According to the present data, either the use of iodized salt or daily cow’s milk consumption suffice alone to reach a UIC within the adequate range, but that is still a borderline adequacy. Only combining the two sources of iodine guaranteed a higher UIC, fully adequate, but still below the threshold for a ‘more than adequate’ iodine supply. Students who neither drink cow’s milk on a regular basis nor use iodized salt have lower UIC in our series. This data combined, together with the fall in UIC from USI campaign introduction (from 2001 (median UIC of 140 μg/L) [6] to 2012 (median UIC of 81 μg/L) [11]) with the reduction of milk use, suggest that the legislator should pay attention to dairy products consumption data, when the iodine prophylaxis campaign.

We also confirmed the role of cow’s milk suggested in our previous reports [11,21], which is due to the diet of dairy cows being enriched with several minerals, including iodine [22].

The percentage of pupils with a UIC < 50 μL is higher in females than in males (30.3% versus 23.6%), with a superimposable iodized salt consumption in males and females, but a less frequent use of milk in the latter. Adolescent females might be induced to reduce their milk and dairy product consumption due to a misconception that they are fattening [23]. Recent observational studies have shown, instead, that milk and dairy products can be beneficial in lowering blood pressure and low-density lipoprotein cholesterol, and in preventing tooth decay, diabetes, cancer, and obesity [24,25]. UIC in females is adequate but borderline, while in males it complies much more with WHO criteria. Females had a lower intake of milk compared to males which may have contributed to their lower median UIC when compared to males. Milk surprisingly resulted, at logistic regression, as much more able to influence UIC than iodized salt use. It is likely that in our population of borderline iodine sufficiency and still suboptimal iodized salt spread, milk frequency use is able to make the difference in iodine status. If milk consumption continues to decrease, the use of iodized salt may become more important in the future.

Iodized salt use resulted as quite unchanged when its use was analyzed based on sex and age range, while milk use frequency percentages were much more affected by sex. Eleven to twelve-years-old and 15–16-years-old females resulted as iodine insufficient, although the small sample sizes limited our ability to further analyze this data.

Whatever their reasons, females were confirmed as being poor milk consumers in this latest survey also, when we recorded a median UIC in female SAC of 103 μg/L, barely enough to be considered an adequate iodine supply. This situation warrants attention because these girls could be future mothers and, to prevent iodine deficiency and its consequences, they should be informed that they need a higher iodine intake—not only in pregnancy and breastfeeding, but also prior to conception. Their iodine intake should be raised to 150 μg/day, according to recent ATA guidelines [26].

It is worth noting how milk products are also able to influence UIC: regular consumption of cheese or yogurt (more than once a week) proved effective in achieving an adequate median UIC even in students not using iodized salt. The present study is the first in Italy to find a significant association between cheese consumption and UIC. Unfortunately, our survey did not collect information on the type of cheese the SAC ate. In a previous report by our group we assessed the iodine content of milk commercially available in Veneto region. Based on that data, the median and mean iodine values of fresh and long-life commonly commercially available cow milk samples were 262 and 264 μg/L, respectively [11]. Unfortunately, to our knowledge, little scientific information is available about the iodine concentrations in Italian cheeses. Some papers have investigated iodine from a cheese production perspective. It is well known that a large proportion of the iodine in milk is lost in the whey during the cheese-making process, but the loss of moisture during the cheese curdling and ripening processes raises the iodine concentration in cheese from 1.7 [27] to 2.6 times that of milk [28]. Haldimann et al. [29] studied the diffuse migration of iodine in four different types of hard cheese after immersion in brine supplemented with iodized salt in the form of potassium iodine. They demonstrated that salting cheeses in brine supplemented with iodized salt led to a significant 10-fold increase in the average iodine content of cheeses. As for the contribution of milk products to iodine intake in our region, it is worth mentioning that a study of ours in 2001 (before the iodine prophylaxis campaign) on a limited group of SAC living in north-east Italy, documented an adequate median UIC (140 μg/L) although iodized salt was used by less than 30% of participants [6]. With the limitation of the small sample of SAC recruited in 2001, we speculate that dairy products may have contributed considerably to this result. In fact, major changes seen in dietary behavior in our region from 2001 to 2019 have included a gradual decrease in the numbers of SAC interviewed reportedly consuming cow’s milk, yogurt, and cheese on a regular basis (69% to 46.7% for milk; from 48% to 28.6% for yogurt; and from 65% to 44.2% for cheese) [6,11].This has providentially coincided with an increasing use of iodized salt [6]. This picture is in line with other reports of a decline in milk consumption in high-income countries [30]. In 2014, for instance, Dror et al. [12] reported a fall in milk and dairy product consumption by children and adolescents of developed countries. The authors identified some of the possible variables involved as age, sex, parental influence, replacement with other beverages, and changing dietary patterns.

The SAC’s awareness of legislation concerning iodine deficiency documented in this report has not changed when compared to the previous survey, undergone in 2012 (42.5% versus 45%) [21]. In our survey, awareness of the legislation to deal with iodine deficiency was associated with a greater propensity to use iodized salt, thus confirming the importance and efficacy of educational intervention in schools and other institutions to improve iodine status in the population [9].

Our study has many limitations. We included in the survey many middle schools selected by our local hygiene service, that should be representative of all Veneto region. However, since not schools are included, a possible bias may come from the representativeness of our series, mostly in the 15–16- and 11–12-year old age range. Moreover, the number of pupils included in the study was lower than expected, since not all students complied with the inclusion criteria.

Finally, based on our data, adolescent females should be the main target of the next iodine prophylaxis campaigns.

## Figures and Tables

**Table 1:** Median urinary iodine concentration (UIC) (μg/L) and % of pupils with a UIC < 100 μg/L, iodized salt use and regular milk consumption (daily or more) according to sex and age group.

		Total	11–12 Years	13–14 Years	15–16 Years	*p* *
			(n = 100/474, 13.4%)	(n = 624/747, 83.6%)	(n = 23/474, 3%)	
	**Median (μg/L) (IQR)**	111.0	112.4	111.6	130.8	0.98
		(55.9–198.0)	(37.8–194.7)	(48.0–188.4)	(41.7–205.5)	
	**UIC < 50 μg/L n (%)**	193/747	32/100	154/624	7/23	0.81
		(26.0)	(32.0)	(24.8)	(30.4)	
**M**	**Median (μg/L)**	121.0	130.8	114.0	130.8	0.79
	**(IQR)**	(56.4–199.2)	(56.1–2262)	(55.2–199.5)	(77.1–188.1)	
	**UIC < 50 μg/L n (%)**	97/411	12/54	82/342	3/15	0.82
		(23.6)	(22.2)	(23.9)	(20.0)	
	**Iodized salt use n (%)**	336/411	45/54	277/342	12/15	0.66
		(81.8)	(83.3)	(80.9)	(80.0)	
	**Regular milk use n (%)**	221/411	24/54	189/342	8/15	0.42
		(54.0)	(44.4)	(55.3)	(53.3)	
**F**	**Median (μg/L)**	103.2	85.2	106.8	55.8	0.53
	**(IQR)**	(37.2–174.0)	(36.0–171.6)	(41.1–176.1)	(40.8–248.4)	
	**UIC < 50 μg/L n (%)**	102/336,	20/46	78/282	4/8	0.22
		(30.3)	(42.5)	(27.6)	(50.0)	
	**Iodized salt use n (%)**	279/336	41/46	230/282	8/8	0.26
		(83.0)	(89.0)	(81.5)	(100.0)	
	**Regular milk use n (%)**	126/336	21/46	100/282	5/8	0.14
		(38.0)	(45.6)	(35.4)	(62.5)	

UIC = urinary iodine concentration, F: females, M: males; * *p* by Kruskal–Wallis test for median UIC values and by chi-squared test for UIC<50 μg/L and for iodized salt and milk use.

**Table 2:** School-age children’s reported eating habits and urinary iodine concentration (UIC) adequacy (on the right, divided for the percentage (%) of samples with a UIC ≥ 100 μg/L, between 50 μg/L and < 100 μg/L and < 50 μg/L) and the corresponding median UIC in the whole sample (on the left) according to iodized salt use and food milk, yogurt, and cheese consumption frequency.

Product		Total	Median UIC (μg/L) (Interquartile Range)	*p* **	Percentage of UIC < 50 ug/L 193/747 (26%)	Percentage of UIC ≥ 50 and < 100 ug/L 138/747 (18%)	Percentage of UIC ≥ 100 ug/L 416/747 (56%)	*p* *
**Iodized salt**	**yes**	610/747 (81.6%)	117.0 (54.0–192.0)	0.01	147/610 (24.0%)	112/610 (18.4%)	351/610 (57.6%)	0.02
	**no**	137/747 (18.4%)	90.0 (36.6–174.6)		51/137 (37.2%)	23/137 (16.7%)	63/137 (45.9%)	
**Cow’s milk**	**regularly**	349/747 (47%)	132.0 (72.0–198.9)	< 0.01	68/349 (19.6%)	56/349 (16.1%)	225/349 (64.3%)	< 0.01
	**occasionally**	398/747 (53%)	96.0 (36.0–177.3)		127/398 (31.9%)	77/398 (19.3%)	194/398 (48.8%)	
**Cow’s milk**	**1**	143/747 (19.12%)	96.0 (36.0–162.6)	< 0.01	45/143 (31.1%)	28/143 (19.7%)	70/143 (49.2%)	< 0.01
	**2**	255/747 (34.1%)	94.8 (36.0–183.6)		82/255 (32.3%)	49/255 (19.1%)	124/255 (48.5%)	
	**3**	321/747 (43%)	130.8 (69.6–192.6)		65/321 (20.3%)	53/321 (16.6%)	203/321 (63.2%)	
	**4**	28/74 (3.8%)	223.2 (115.8–273.0)		3/28 (11.5%)	3/2 (11.5%)	22/28 (76.9%)	
**Yogurt**	**1**	205/747 (27.4%)	110.4 (40.2–188.4)	0.22	60/205 (29.3%)	32/205 (15.4%)	113/205 (55.3%)	0.26
	**2**	329/747 (44%)	109.2 (44.4–183.6)		89/329 (27.2%)	58/329 (17.5%)	182/329 (55.3%)	
	**3**	213/747 (28.6%)	126.6 (62.4–195.6)		43/213 (20.4%)	45/213 (20.9%)	125/213 (58.7%)	
**Cheese**	**1**	89/747 (12%)	87.6 (39.6–165.6)	0.16	26/89 (29.3%)	25/89 (28%)	38/89 (42.7%)	0.02
	**2**	328/747 (43.9%)	112.8 (39.3–199.5)		96/328 (29.2%)	51/328 (15.6%)	181/328 (55.21%)	
	**3**	330/747 (44.1%)	116.4 (57.9–184.8)		74/330 (22.4%)	57/330 (17.2%)	199/330 (60.4%)	
**Beef**	**1**	33/747 (4.4%)	135.0 (48.0–244.8)	0.19	9/33 (26.7%)	3/33 (10%)	21/33 (63.3%)	0.52
	**2**	395/747 (52.9%)	116.4 (54.9–194.4)		230/395 (58.2%)	69/395 (17.5%)	96/395 (24.3%)	
	**3**	319/747 (42.7%)	105.6 (44.7–176.4)		169/319 (53.0%)	61/319 (19.1%)	89/319 (27.9%)	
**Chicken**	**1**	10/747 (1.3%)	130.8 (75.6-257.4)	0.56	2/10 (22.2%)	1/10 (11.1%)	7/10 (66.7%)	0.56
	**2**	410/747 (54.9%)	114.0 (51.6–188.0)		100/410 (24.4%)	75/410 (18.3%)	235/410 (57.3%)	
	**3**	327/74 (43.8%)	110.4 (40.8–183.9)		93/327 (28.5%)	57/327 (17.4%)	177/327 (54.1%)	
**Eggs**	**1**	78/747 (10.4%)	96.0 (40.8–192.6)	0.77	23/78 (29.2%)	17/78 (22.2%)	38/78 (48.6%)	0.68
	**2**	591/747 (79.2%)	114.0 (48.0–186.0)		152/591 (25.7%)	102/591 (17.3%)	337/591 (57.0%)	
	**3**	78/747 (10.4%)	112.8 (36.6–112.4)		22/78 (27.8%)	12/78 (15.3%)	44/78 (56.9%)	
**Fish**	**1**	156/747 (20.9%)	110.4 (39.6–195.6)	0.89	47/156 (30.1%)	26/156 (16.7%)	83/156 (53.2%)	0.82
	**2**	524/747 (70.1%)	112.8 (39.8–188.4)		131/524 (25.0%)	96/524 (18.4%)	297/524 (56.6%)	
	**3**	67/747 (9%)	121.2 (48.0–176.4)		18/67 (27.4%)	11/67 (16.1%)	38/67 (56.5%)	
**Chips**	**1**	107/747 (14.3%)	115.2 (40.8–186.0)	0.77	28/107 (26.2%)	18/107 (16.8%)	61/107 (57.0%)	0.98
	**2**	512/747 (68.5%)	114.0 (48.0–191.7)		135/512 (26.3%)	90/512 (17.6%)	287/512 (56.1%)	
	**3**	128/747 (17.2%)	110.4 (49.2–176.4)		32/128 (25.0%)	25/128 (19.5%)	71/128 (55.5%)	
**Pizza**	1	17/747 (2.3%)	85.2 (36.0-125.4)	0.28	5/17 (29.4%)	5/17 (29.4%)	7/17 (41.2%)	0.38
	**2**	681/747 (91.1%)	112.8 (48.0–188.4)		178/681 (26.1%)	121/681 (17.8%)	382/681 (56.1%)	
	**3**	49/747 (6.6%)	112.8 (45.0–188.1)		13/49 (26.7%)	5/49 (11.1%)	31/49 (62.2%)	
**Cold Cuts**	**1**	49/747 (6.5%)	115.2 (47.1–214.5)	0.87	13/49 (26.5%)	8/49 (16.3%)	28/49 (57.2%)	0.99
	**2**	330/747 (44.2%)	110.4 (48.0–189.3)		88/330 (26.7%)	59/330 (17.9%)	183/330 (55.4%)	
	**3**	368/747 (49.3%)	112.8 (48.0–184.8)		94/368 (25.6%)	66/368 (17.9%)	208/368 (56.5%)	
**Canned Food**	1	374/747 (50.0%)	114.0 (45.3–188.7)	0.58	101/374 (27.0%)	58/374 (15.5%)	215/374 (57.5%)	0.32
	**2**	329/747 (44.1%)	110.4 (49.8–187.8)		82/329 (25.0%)	64/329 (19.3%)	183/329 (55.7%)	
	**3**	44/747 (5.9%)	84.6 (40.8–145.8)		12/44 (27.5%)	12/44 (27.5%)	20/44 (45%)	
**Soy Milk**	**regularly**	738/747 (98.8%)	119.4 (44.8–278.5)	0.81	193/738 (26.1%)	134/738 (18.2%)	411/738 (55.7%)	0.90
	**occasionally**	9/747 (1.2%)	110.4 (104.4–122.4)		2/9 (25.0%)	1/9 (12.5%)	6/9 (62.5%)	
**Soft Drinks**	**1**	331/747 (44.3%)	104.4 (44.4–183.6)	0.19	91/331 (27.5%)	65/331 (19.6%)	175/331 (52.9%)	0.40
	**2**	382/747 (51.2%)	122.4 (51.6–193.5)		93/382 (24.4%)	64/382 (16.7%)	225/382 (58.9%)	
	**3**	34/747 (4.5%)	110.4 (36.0–174.9)		12/34 (35.5%)	4/34 (12.9%)	18/34 (51.6%)	

Milk and soy milk: regularly = daily or more; occasionally = less than once a day); milk 1 = no consumption; 2 = occasional consumption; 3 = 1 cup a day (at least 200 mL/d); 4 = more than 1 cup a day. For other foods, intake was scored as: 1 = rarely or never; 2 = once a week; or 3 = more than once a week. *p* ** by Mann–Whitney test for iodized salt (yes/no), cow’s milk and soy milk frequency (regularly/occasionally), and by Kruskal–Wallis test for milk frequency (1/2/3/4) and for yogurt, cheese, beef, chicken, eggs, fish, chips, pizza, cold cuts, canned food, and soft drinks frequency (1/2/3). *p* * by chi-squared test.
